# Employment is a critical mental health intervention

**DOI:** 10.1017/S2045796020000906

**Published:** 2020-11-05

**Authors:** Robert E. Drake, Michael A. Wallach

**Affiliations:** 1 Westat, Lebanon, New Hampshire, USA; 2 Department of Psychology and Neuroscience, Duke University, Durham, North Carolina, USA

**Keywords:** Community mental health, evidence-based psychiatry, health service research, psychiatric services

## Abstract

Abstract employment is critically important in mental health care. Unemployment worsens mental health and gaining employment can improve mental health, even for people with the most serious mental illnesses. In this editorial, we argue for a new treatment paradigm in mental health that emphasises employment, because supported employment is an evidence-based intervention that can help the majority of people with mental health disability to succeed in integrated, competitive employment. Unlike most mental health treatments, employment engenders self-reliance and leads to other valued outcomes, including self-confidence, the respect of others, personal income and community integration. It is not only an effective short-term treatment but also one of the only interventions that lessen dependence on the mental health system over time.

Like most of us today, people in early Egyptian civilization 5000 years ago organised their lives around jobs and families (Vischak, 2014). Physicians since the time of Hippocrates (circa 400 BCE) have recognised that work leads to a healthy life. Research in the modern era robustly confirms that employment improves health and unemployment leads to deterioration of health (Rueda *et al*., 2012). The same applies to people with mental health conditions. Researchers and policy makers now agree that employment is both a critical health intervention and a meaningful outcome for people with serious mental disorders such as schizophrenia, bipolar disorder and depression (Knapp and Wong, 2020). This recognition follows patients' own expressed goals as well as actual work outcomes. People with even the most serious mental disorders report a higher quality of life, greater self-esteem and fewer psychiatric symptoms when they are employed (Luciano *et al*., 2014).

Yet acceptance that employment is a key determinant of mental health and should be a central goal of mental health treatment has not translated into policy and increased the employment rate of people with serious mental disorders. In fact, only about 10–15% of these individuals are actually working in the USA, the UK, and other high-income countries (Drake, 2020). The prevailing biomedical paradigm has not increased the employment rate, because medications often reduce symptoms without improving social functioning (Percudani *et al*., 2004). However, we know that supported employment could help the majority of people with serious mental health disorders attain competitive employment (Metcalfe *et al*., 2018).

We, therefore, argue for a new mental health treatment paradigm asserting that helping people with employment should be a standard mental health intervention. The mental health field needs a new treatment paradigm that incorporates employment for several reasons. First, employment improves the mental health and wellbeing of people with serious mental disorders, including improved self-esteem, symptom control, quality of life, social relationships and community integration, without harmful side effects (Drake *et al*., 2013). No other mental health intervention consistently shows these important benefits.

Second, an effective approach to help people achieve competitive employment that now exists. Individual Placement and Support (IPS supported employment) has emerged over the last 30 years as an evidence-based approach, based on 28 randomised controlled trials (Bond *et al*., 2020). It enables about 60% of unselected participants with serious mental disorders to succeed in competitive employment – two to three times better than other employment interventions (Frederick and VanderWeele, 2019). IPS has been successful in many countries and for people with a range of disorders (Drake, 2020).

Third, helping people with mental health disabilities to succeed in competitive employment is an ethical and legal imperative. The great majority of people with serious mental disorders desire employment as a primary treatment goal (Wescott *et al*., 2015) and legal standards in several high-income countries mandate creating opportunities for employment (e.g. the Americans with Disabilities Act of 1990).

Finally, supported employment is a cost-effective and possibly cost-saving intervention because it decreases the use of psychiatric hospitals and overall mental health spending (Knapp *et al*., 2013). Supported employment is a relatively inexpensive intervention (Latimer *et al*., 2004) and employment leads to steady reductions in mental healthcare costs over at least 10 years (Bush *et al*., 2009).

Medical costs across high-income countries continue to spiral upward, in part because the prevailing treatment paradigm involves greater use of specialists, expensive technology and new medications. In psychiatry, several cycles of promised biomedical breakthroughs over the last 100 years have produced expensive failures rather than better outcomes (Harrington, 2019). By and large, the beneficiaries have been the pharmaceutical and biomedical industries, not the people with serious mental disorders.

A new paradigm should emphasise social function, especially the centrality of employment as an effective treatment that more directly corresponds to what people want. Employment enhances the quality of life without curing mental disorders, but as America's leading psychiatric advocate, Dr Patricia Deegan, has argued for decades, people with mental disorders view ‘recovery’ as a meaningful, active, functional life, not as a complete absence of symptoms (Deegan, 1988). People can learn to tolerate and cope with symptoms if they have a life that they consider valuable.

Employment is a humble, unglamorous, but achievable goal. Medications, psychotherapies, skills training and other interventions promise to improve functioning but often fail because they assume a stepwise transfer to the criterion outcome. As demonstrated consistently in education research, interventions that are closest to a clear, unambiguous criterion of interest are more likely to enhance that criterion than ones that are indirect (Wallach, 1976). In mental health, effective approaches help people to find employment, housing, education, or friends directly by providing the minimal supports they want and need. Thus, while pre-employment interventions rarely lead to competitive employment, supported employment directly helps people with mental health conditions to find jobs they choose and supports them to succeed in the jobs.

Defining supported employment as a mental health intervention would have numerous practical ramifications as well. If 50% rather than 10% of the millions of adults with such disorders were in the workforce, employers would have access to a needed supply of good workers (according to feedback from employers), communities would have fewer of the social problems that accompany unemployment, people would be paying taxes rather than needing social services and direct social contacts in the workplace would lessen stigma against people with mental illness (IPS Grow, 2018).

Defining supported employment as a core mental health intervention could lead to a sensible service package for people with serious mental disorders. In the USA, current piecemeal funding for specific interventions fragments services interferes with efforts to collaborate across disciplines and results in non-integrated care. Expecting people to visit multiple agencies and clinics with different procedures, forms, staff, agendas, interventions and recovery messages produce high rates of dropout – a result of overwhelming bureaucracy rather than noncompliance. People who have chronic mental illnesses need an integrated package of services delivered by a collaborative team and individualised for their particular needs and goals (Drake *et al*., 2020). A team-based approach is particularly helpful to people with serious mental disorders because cognitive problems are a common aspect of their disorders. Funding should pay for a service package that is coherent, accessible and aimed at the individual's functional goals. For example, a young person who develops schizophrenia and lives with family typically needs several, integrated services: medical care, medications, supported employment/education, family psychoeducation and support for illness management.

Unlike most mental health treatments, employment engenders self-reliance and leads to other valued outcomes, including self-confidence, the respect of others, personal income and community integration. It is not only an effective short-term treatment but also one of the only interventions that lessen dependence on the mental health system over time. Virtually all books and self-reports on personal recovery feature employment – work of some kind – as a central step. The goals of treatment should be, as Freud put it a century earlier, to work and to love. These are realistic goals, not usually achieved by biomedical interventions, but practical and feasible using evidence-based, non-technical interventions such as supported employment.

A meaningful life is not necessarily a life free from psychiatric symptoms. It depends on whether the symptoms preclude the patient's overall flourishing. And that, in turn, depends on whether the symptoms are the focus or a surmountable distraction from the patient's own higher priorities in life.

The contrast with expensive biomedical interventions should be obvious. Pat Deegan described her recovery as follows: Her doctor told her she was a treatment success when she was heavily medicated, unable to focus her thinking, and smoking cigarettes in front of a television all day – but she wanted a full life. She learned to live with fewer medications and some symptoms, returned to college and graduate school and became a successful psychologist, wife and mother (Deegan, 1998). People with mental illness typically express more modest but equally meaningful goals. They want a safe apartment; a part-time job; and the chance to meet people, have friends, contribute to society and participate in community life that comes with a job and a modest income. They also value the secondary benefits – a positive identity, structure to the day, enhanced self-esteem, friends at work, less interaction with the mental health system and reduced personal and social stigma – gains that do not usually follow hospitalisation, polypharmacy or involuntary treatment.

Treatment must aim toward more than suppressing symptoms. The opportunity to pursue a meaningful life is a fundamental human right. We can easily do better by including supported employment as an essential part of treatment.
